# Willis covered stent treatment for blood blister-like aneurysm: A meta-analysis of efficacy and safety

**DOI:** 10.3389/fneur.2022.1101625

**Published:** 2023-02-21

**Authors:** Jiahe Tan, Rui Song, Siyue Luo, Wenqiao Fu, Jun Su, Zhaohui He

**Affiliations:** ^1^Department of Neurosurgery, First Affiliated Hospital of Chongqing Medical University, Chongqing, China; ^2^Key Laboratory of Molecular Biology for Infectious Diseases (Ministry of Education), Department of Infectious Diseases, Institute for Viral Hepatitis, Second Affiliated Hospital of Chongqing Medical University, Chongqing, China; ^3^Clinical Medicine, Second Clinical College of Chongqing Medical University, Chongqing, China

**Keywords:** covered stent, blood blister-like aneurysm, efficacy, safety, meta-analysis

## Abstract

**Background:**

Blood blister-like aneurysm (BBA) is a rare and special type of intracranial aneurysm with extremely high rates of rupture, morbidity, mortality, and recurrence. Willis Covered Stent (WCS) is a new device that is specifically designed for the treatment of intracranial complex aneurysms. However, the efficacy and safety of WCS treatment for BBA remain controversial. Thus, a high level of evidence is required to prove the efficacy and safety of WCS treatment.

**Methods:**

A systematic literature review was performed using a comprehensive literary search in Medline, Embase, and Web of Science databases to identify studies related to WCS treatment for BBA. A meta-analysis was then conducted to incorporate the efficacy and safety outcomes, including intraoperative situation, post-operative situation, and follow-up data.

**Results:**

Eight non-comparative studies containing 104 patients with 106 BBAs met the inclusion criteria. In the intraoperative situation, the technical success rate was 99.5% [95% confidence interval (CI), 0.958, 1.000], the complete occlusion rate was 98.2% (95% CI, 0.925, 1.000), and the side branch occlusion rate was 4.1% (95% CI, 0.001, 0.114). Vasospasm and dissection occurred in 9.2% (95% CI, 0.000, 0.261) and 0.1% (95% CI, 0.000, 0.032) of the patients, respectively. In the post-operative situation, the rebleed and mortality rates were 2.2% (95% CI, 0.000, 0.074) and 1.5% (95% CI, 0.000, 0.062), respectively. In the follow-up data, recurrence and parent artery stenosis occurred in 0.3% (95% CI, 0.000, 0.042) and 9.1% (95% CI, 0.032, 0.168) of the patients, respectively. Ultimately, 95.7% (95% CI, 0.889, 0.997) of the patients had a good outcome.

**Conclusions:**

Willis Covered Stent could be effectively and safely applied for BBA treatment. The results provide a reference for clinical trials in the future. Well-designed prospective cohort studies must be conducted for verification.

## Introduction

Blood blister-like aneurysm (BBA), a name derived from its bright red, blood-blistered appearance under direct vision, refers to the aneurysm located at the non-branching sites of the anterior or the dorsal wall of the intracranial internal carotid artery and accounts for 1% of all intracranial aneurysms (1, 2). Owing to its features of a histologically fragile wall and a morphologically wide neck, BBA is prone to rupture, may lead to subarachnoid hemorrhage, and has high morbidity, mortality, and recurrence rates (3). Hence, its treatment is extremely challenging.

With the improvement in endovascular treatment techniques and the development of new materials, especially the flow diverter (FD) and the covered stent, BBA treatment has changed from “embolization of aneurysm” to “repair of parent artery” (4). However, optimal management remains controversial.

Willis Covered Stent (WCS), which was developed in China, is a new device that is specifically designed for the treatment of intracranial complex aneurysms. By isolating the aneurysm cavity and the parent artery, this “China Option” could reconstruct the anatomy and restore the normal hemodynamics of the parent artery to treat aneurysms (5).

Although WCS has been applied to treat BBA in several clinical centers, its efficacy and safety remain unclear (6). Thus, a high level of evidence is required to prove the efficacy and safety of WCS treatment. Here, the present study aimed to conduct a systematic review of current studies related to the WCS treatment for BBA and a meta-analysis to incorporate the outcomes of efficacy and safety.

## Materials and methods

The review was prospectively registered with the International Prospective Register of Systematic Reviews (PROSPERO) database (CRD42022377151).

### Literature search

Studies related to WCS treatment for BBA were identified after a comprehensive literature search in Medline, Embase, and Web of Science databases until November 14, 2022. Search terms included “blood blister like aneurysm,” “blood blister-like aneurysm,” “blood-blister-like aneurysm,” “blister like aneurysm,” “blister-like aneurysm,” “blood blister aneurysm,” “blood-blister aneurysm,” “blister aneurysm,” “BBA,” “covered stent,” “willis covered stent,” and “WCS” in “AND” and “OR” combinations. The year of publication and language were not restricted.

### Inclusion and exclusion criteria

The inclusion criteria were as follows: (1) non-comparative studies analyzing the efficacy and safety of WCS treatment for BBA; (2) clear definition and same diagnostic criteria of BBA; (3) use of only WCS treatment performed by experienced interventional physicians, and the perioperative management of patients must be standard, especially the antiplatelet therapy strategy; (4) single study including more than two patients; and (5) studies reporting initial data of the outcomes, including intraoperative situation, postoperative situation, and follow-up data. The exclusion criteria were as follows: (1) repetitive articles or cohorts, (2) co-treatment of BBA, (3) lack of initial data, and (4) studies other than the non-comparative study. Studies were independently selected by two authors (Tan and Song) on the basis of the mentioned criteria. Disagreements were resolved by consensus with a third author (Luo).

### Data extraction

Data included baseline characteristics (such as the first author, publication year, number of patients and BBAs, mean age, sex, and mean BBA size); intraoperative situation (number of technical successes, aneurysm complete occlusion, side branch occlusion, vasospasm, and dissection); postoperative situation (number of rebleeds and mortality); and follow-up data (duration, number of patients, recurrence, parent artery stenosis, and good outcome). The evaluation indicators for efficacy were technical successes, aneurysm complete occlusion, recurrence, and good outcome. The evaluation indicators for safety were side branch occlusion, vasospasm, dissection, rebleed, mortality, and parent artery stenosis. Technical success was defined as WCS successfully implanted in the parent artery of BBA. Occlusion, vasospasm, dissection, recurrence, stenosis, and rebleed were confirmed by the imaging examination [digital subtraction angiography, computed tomography (CT), computed tomography angiography (CTA), or magnetic resonance angiography (MRA)]. A good outcome was defined as a modified Rankin Scale score (mRS) of 0–2. Data were extracted independently by two authors (Tan and Song), and disagreements were resolved by consensus with a third author (Luo).

### Quality assessment

The methodological quality of each study was independently evaluated by two authors (Tan and Song), according to the Agency for Healthcare Research and Quality (AHRQ) 11-item checklist (7). One point was given for “YES” of each of the following criteria: (1) define the source of information (survey, record review), (2) list inclusion and exclusion criteria for exposed and unexposed subjects (cases and controls) or refer to previous publications, (3) indicate time period used for identifying patients, (4) indicate whether or not subjects were consecutive if not population-based, (5) indicate if the evaluators of subjective components of study were masked to other aspects of the status of the participants, (6) describe any assessments undertaken for quality assurance purposes (e.g., test/retest of primary outcome measurements), (7) explain any patient exclusions from analysis, (8) describe how confounding was assessed and/or controlled, (9) if applicable, explain how missing data were handled in the analysis, (10) summarize patient response rates and completeness of data collection, and (11) clarify what follow-up, if any, was expected and the percentage of patients for which incomplete data or follow-up was obtained. On the contrary, no point was given to “NO” or “UNCLEAR” answers. The quality of studies was ranked low (≤3 points), moderate (4–7 points), and high (≥8 points). Disagreements were resolved by consensus with a third author (Luo).

### Statistical analysis

Data management, the transformation of the effect size, calculation of the pooled risk difference, and corresponding 95% confidence interval (CI) were performed using the “metaprop” code in the Stata statistical software (version 16.0) (8). For consideration of data compatibility, a random effects model was chosen to pool the event rates for overall outcomes. Heterogeneity across the studies was tested by calculating the I-squared (*I*^2^) statistic (9). *I*^2^ of <50% represented low heterogeneity and *I*^2^ of ≥50% represented high heterogeneity. Forest plots were used to illustrate the results graphically. Owing to limitations caused by the non-comparative nature of the included studies, sensitivity analysis and tests for publication bias were not completed (10).

## Results

Systematic review and meta-analysis were conducted following the Meta-analysis of Observational Studies in Epidemiology (MOOSE) guidelines (11).

### Literature search

**Figure 1:**
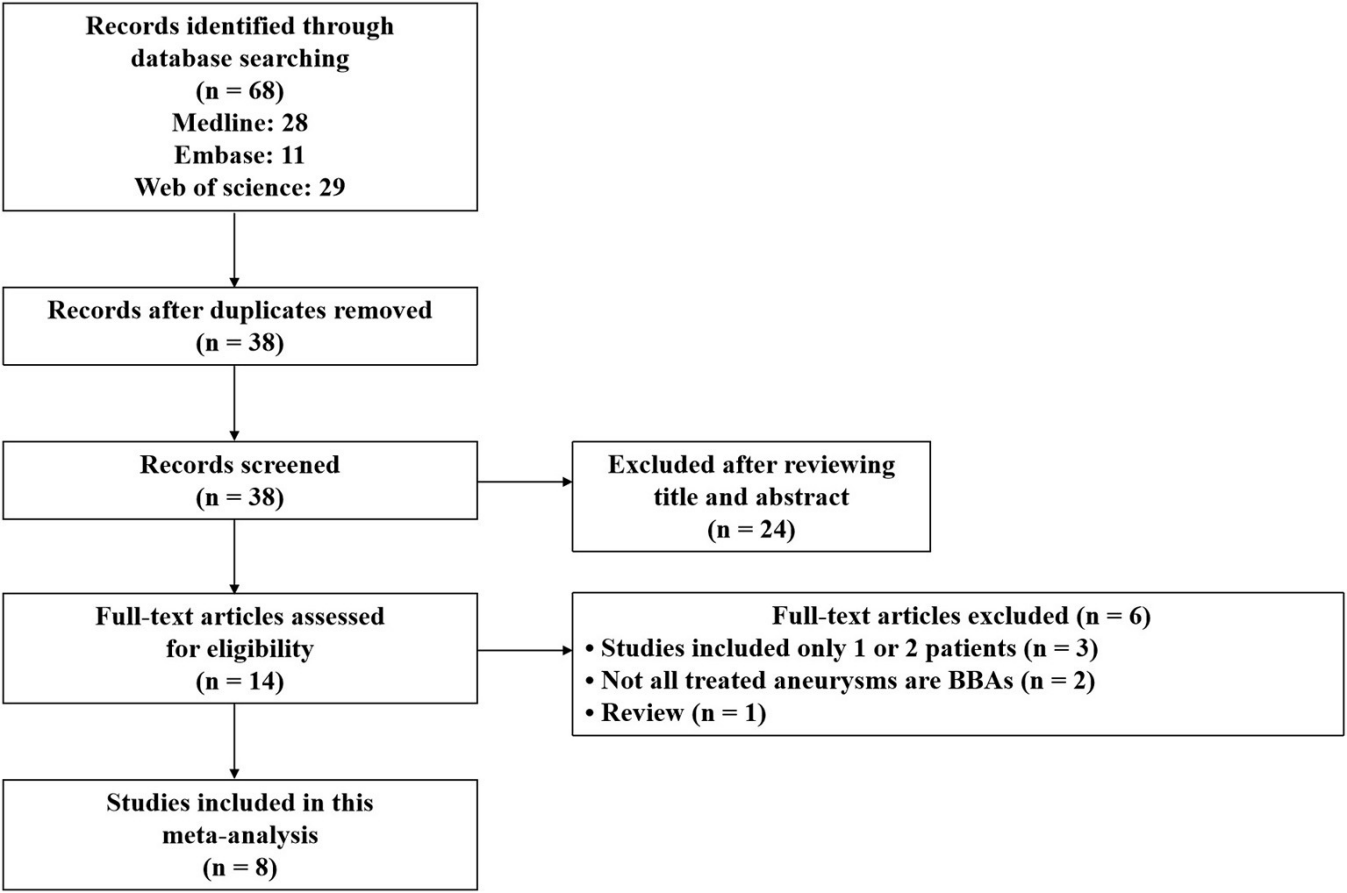
A flowchart of the literature search performed.

After a search of comprehensive literature, 68 records were identified. After the deletion of duplicate records, 38 records remained for the title and abstract review. After studying the title and abstract review, 14 records remained for full-text examination. Of these, three records were excluded because they were single studies that included only one or two patients, and two other records were excluded because not all the treated aneurysms were BBAs. One of the studies was a review. Ultimately, eight non-comparative studies were included in the meta-analysis (12–19). A flow diagram is shown in Figure 1.

### Characteristics of included studies

A total of 104 patients with 106 BBAs were identified in the eight selected studies that were published from 2016 to 2022. The mean age of the patients in the seven selected studies ranged from 49.2 to 54.5 years, the men-to-women ratio in the seven selected studies was 48.3% (28/58), and the mean BBA sizes were clear in the six selected studies. Intraoperative situation, post-operative situation, and follow-up data were described in detail in all of the included studies. The characteristics of included studies are summarized in Table 1.

**Table 1:** Characteristics of included studies.

	**Baseline characteristics**							**Intraoperative situation**	
**References**	**Patients (BBAs)**, ***n***		**Mean age, y**		**Males/ females**, ***n***	**Mean BBA size, mm**	**Technical success**, ***n***	**Complete occlusion**, ***n***	**Side branch occlusion**, ***n***
Wang et al. (12)	8/8		49.2		1/7	NA	7	7	0
Fang et al. (13)	13/15		52.8		7/6	2.3 * 3.3	13	12	3
Gu et al. (14)	20/20		50.6		5/15	3.3 * 5.1	19	15	0
Liu et al. (15)	14/14		54.5		9/5	4.3 * 2.8	14	14	2
Liu et al. (16)	7/7		53.9		1/6	5.7 * 6.0	7	7	0
Chang et al. (17)	18/18		NA		NA	3.5 * 4.3	18	18	0
Fang et al. (18)	16/16		50.6		5/11	3.3 * 2.8	16	16	1
Qi et al. (19)	8/8		50.3		0/8	NA	8	8	1
	**Intraoperative situation**		**Post-operative situation**				**Follow-up data**		
**References**	**Vasospasm**, ***n***	**Dissection**, ***n***	**Rebleed**, ***n***	**Mortality**, ***n***	**Patients**, ***n***	**Duration, m**	**Recurrence**, ***n***	**Parent artery stenosis**, ***n***	**Good outcome**, ***n***
Wang et al. (12)	0	0	0	0	7	3–6	0	1	7
Fang et al. (13)	0	0	0	0	13	4–6	0	2	13
Gu et al. (14)	4	0	1	1	17	3–36	0	2	14
Liu et al. (15)	8	0	1	1	13	3–15	2	1	13
Liu et al. (16)	0	0	0	0	7	6–10	0	0	7
Chang et al. (17)	6	1	0	0	17	3–6	0	3	14
Fang et al. (18)	0	0	1	1	13	3–30	0	1	12
Qi et al. (19)	0	0	1	0	8	1–6	0	0	8

y, year; BBA, blood blister-like aneurysm; n, number; mm, millimeter; m, month; NA, not available.

### Quality assessment

All of the included studies were assessed for methodological quality in accordance with the AHRQ checklist. Details of the quality index are presented in Table 2. The scores ranged from 6 to 10 with a mean value of 7.9. The five selected studies were of high quality, and the three selected studies were of moderate quality. No low-quality study was included in the present research.

**Table 2:** AHRQ checklist.

**References**	**(1)**	**(2)**	**(3)**	**(4)**	**(5)**	**(6)**	**(7)**	**(8)**	**(9)**	**(10)**	**(11)**	**Total**
Wang et al. (12)	⋆		⋆	⋆		⋆				⋆	⋆	6
Fang et al. (13)	⋆	⋆	⋆	⋆		⋆				⋆	⋆	7
Gu et al. (14)	⋆	⋆	⋆	⋆	⋆	⋆	⋆			⋆	⋆	9
Liu et al. (15)	⋆	⋆	⋆	⋆		⋆	⋆			⋆	⋆	8
Liu et al. (16)	⋆	⋆	⋆	⋆		⋆		⋆		⋆	⋆	8
Chang et al. (17)	⋆	⋆	⋆	⋆	⋆	⋆	⋆	⋆		⋆	⋆	10
Fang et al. (18)	⋆	⋆	⋆	⋆		⋆	⋆			⋆	⋆	8
Qi et al. (19)	⋆	⋆	⋆	⋆		⋆				⋆	⋆	7

AHRQ, Agency for Healthcare Research and Quality; y, year. (1) Define the source of information (survey, record review). (2) List inclusion and exclusion criteria for exposed and unexposed subjects (cases and controls) or refer to previous publications. (3) Indicate the time period used for identifying patients. (4) Indicate whether or not subjects were consecutive if not population-based. (5) Indicate if evaluators of subjective components of the study were masked to other aspects of the status of the participants. (6) Describe any assessments undertaken for quality assurance purposes (e.g., test/retest of primary outcome measurements). (7) Explain any patient exclusions from the analysis. (8) Describe how confounding was assessed and/or controlled. (9) If applicable, explain how missing data were handled in the analysis. (10) Summarize patient response rates and completeness of data collection. (11) Clarify what follow-up, if any, was expected and the percentage of patients for which incomplete data or follow-up was obtained. ⋆Represents one point.

### Outcomes of BBA treated by WCS

**Figure 2:**
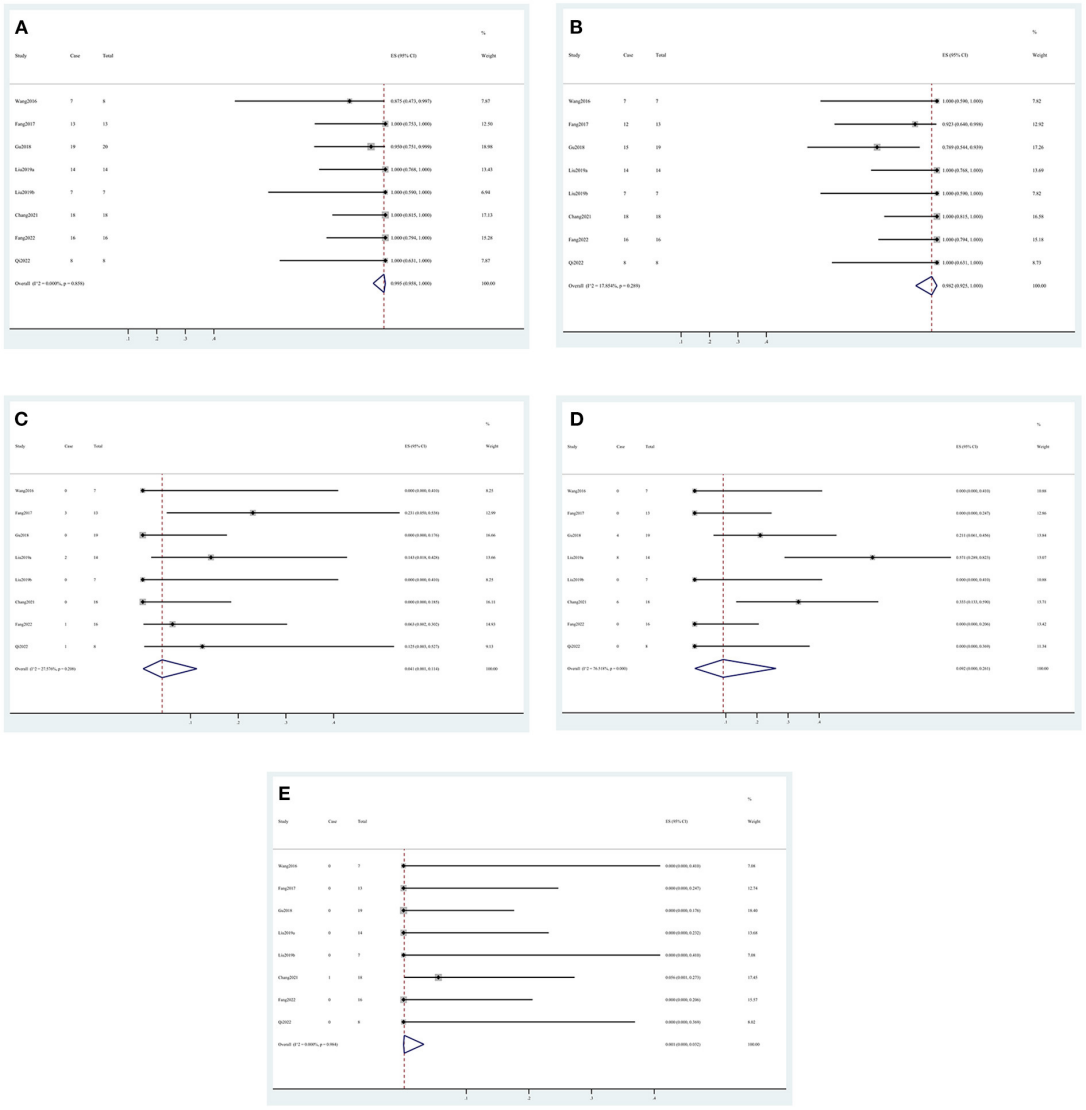
Forest plots of the intraoperative outcome rate. **(A)** Technical success, **(B)** complete occlusion, **(C)** side branch occlusion, **(D)** vasospasm, and **(E)** dissection.

**Figure 3:**
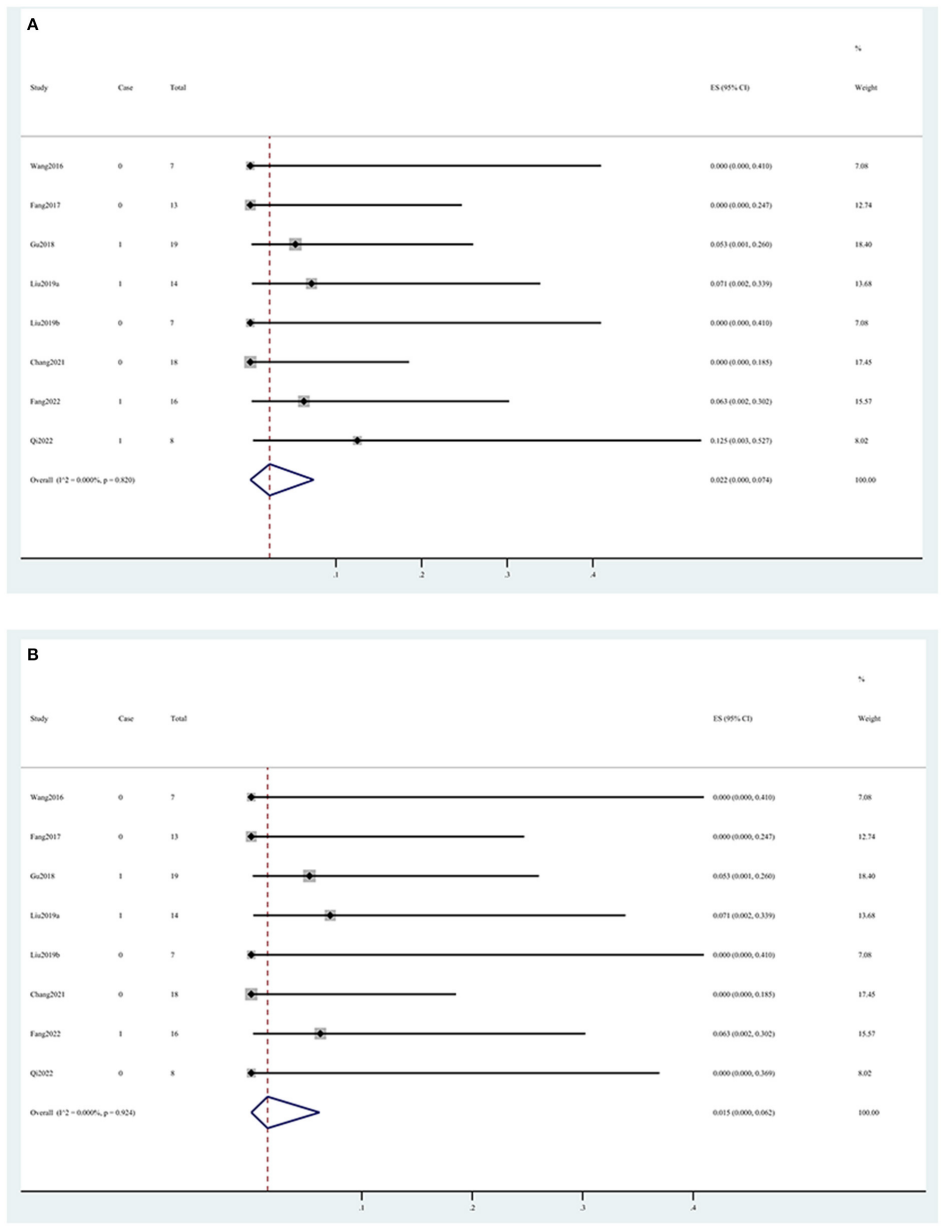
Forest plots of the post-operative outcome rate. **(A)** Rebleed and **(B)** mortality.

**Figure 4:**
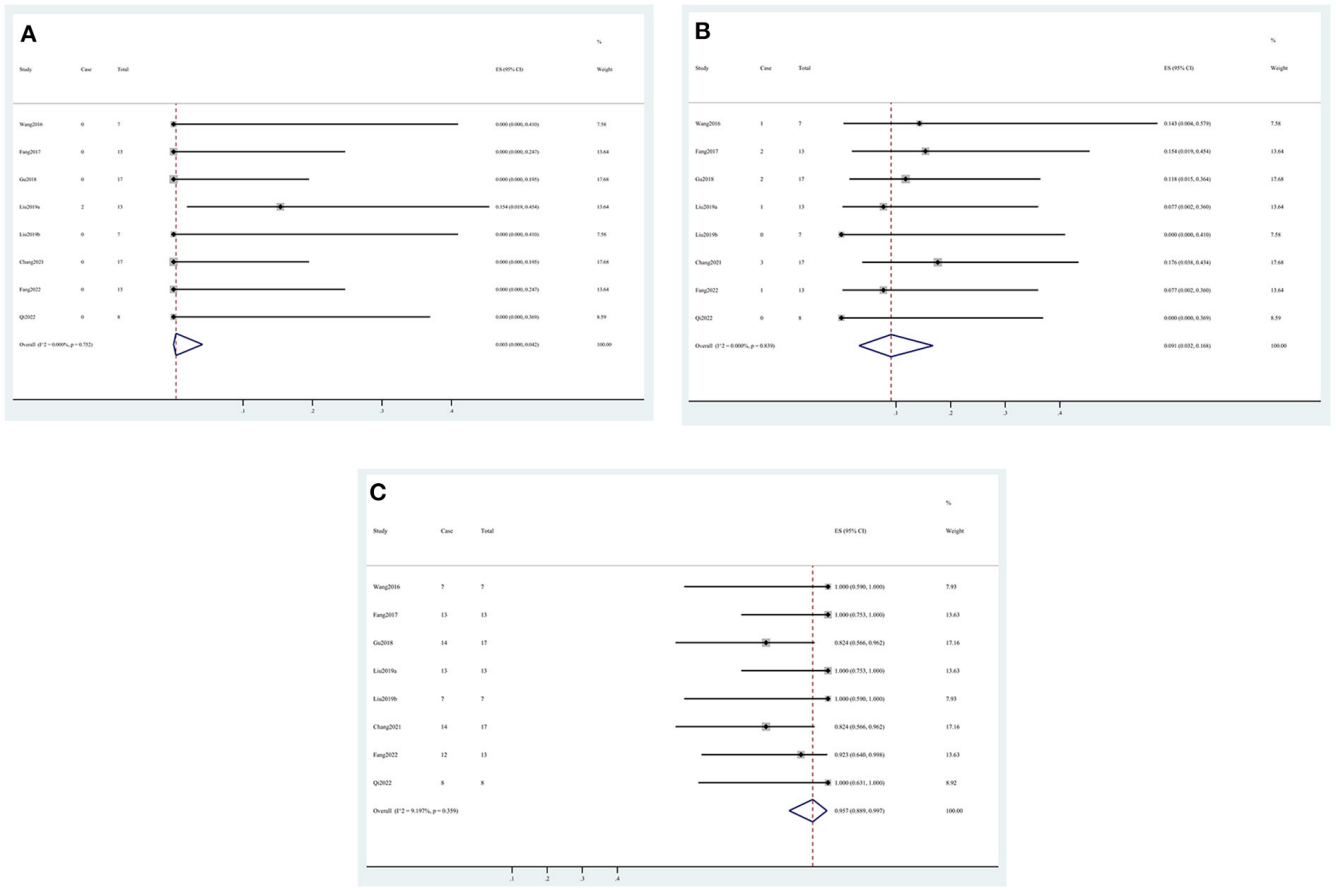
Forest plots of the follow-up outcome rate. **(A)** Recurrence, **(B)** parent artery stenosis, and **(C)** good outcome.

In the intraoperative situation, the technical success rate was 99.5% (95% CI, 0.958, 1.000) (Figure 2A), the complete occlusion rate was 98.2% (95% CI, 0.925, 1.000) (Figure 2B), and the side branch occlusion rate was 4.1% (95% CI, 0.001, 0.114) (Figure 2C). Vasospasm and dissection occurred in 9.2% (95% CI, 0.000, 0.261) (Figure 2D) and 0.1% (95% CI, 0.000, 0.032) (Figure 2E) of the patients, respectively. In the postoperative situation, the rebleed and mortality rates were 2.2% (95% CI, 0.000, 0.074) (Figure 3A) and 1.5% (95% CI, 0.000, 0.062) (Figure 3B), respectively. In the follow-up data, recurrence and parent artery stenosis occurred in 0.3% (95% CI, 0.000, 0.042) (Figure 4A) and 9.1% (95% CI, 0.032, 0.168) (Figure 4B) of the patients, respectively. Ultimately, 95.7% (95% CI, 0.889, 0.997) (Figure 4C) of the patients had a good outcome. The overall outcomes are summarized in Table 3.

**Table 3:** Overall outcomes.

**Outcome**	**Risk difference (95% CI)**	**Raw proportion**	***I^2^* (%)**
Technical success	0.995 (0.958, 1.000)	102/104	0
Complete occlusion	0.982 (0.925, 1.000)	97/102	18
Side branch occlusion	0.041 (0.001, 0.114)	7/102	28
Vasospasm	0.092 (0.000, 0.261)	18/102	77
Dissection	0.001 (0.000, 0.032)	1/102	0
Rebleed	0.022 (0.000, 0.074)	4/102	0
Mortality	0.015 (0.000, 0.062)	3/102	0
Recurrence	0.003 (0.000, 0.042)	2/95	0
Parent artery stenosis	0.091 (0.032, 0.168)	10/95	0
Good outcome	0.957 (0.889, 0.997)	88/95	9

CI, confidence interval; I^2^, I-squared.

## Discussion

To the best of our knowledge, this study is the first systematic review and meta-analysis that has explored the efficacy and safety of WCS treatment for BBA. After the strict screening, eight eligible studies including 106 BBAs in 104 patients were selected. The estimated pooled results showed that WCS had a high success rate of implantation. While ensuring a complete occlusion rate, the WCS treatment exhibited a low rate of intraoperative and post-operative complications, a high BBA cure rate, and a low recurrence rate. The vast majority of patients could obtain a good prognosis.

Owing to its features of a histologically fragile wall and a morphologically wide neck, the BBA treatment remains extremely challenging (3). Microsurgery, endovascular therapy, or a combination of the two comprises the treatment options for BBA. The prevailing views are reconstructing the anatomy and restoring the normal hemodynamics of the parent artery. Endovascular therapy has been recognized as the first choice of BBA treatment. The options are mainly endovascular coiling, multiple overlapping stents with or without coiling, FD, and covered stent (20). Since the first report on WCS treatment for intracranial pseudoaneurysm, studies on WCS treatment for BBA have increased with clinicians' experience and the maturity of technology (21).

In our systematic review, the men-to-women ratio was 28:58 (48.3%). Gonzalez et al. conducted a systematic literature review of BBAs in 2014. A total of 322 patients were evaluated, and the men-to-women ratio was 89:233 (38.2%). The results showed that BBA tended to have female predominance, but no statistical analysis was performed. In the present comprehensive literature review of the BBA, no report has been found on the statistically significant effect of gender on the formation of BBA. Based on the combination of statistics and medical opinion, the possible reason can be that BBA is extremely rare and its pathogenesis is unknown. In addition, the risk factor analysis cannot be completed in studies with small sample sizes (22).

In the meta-analysis, the technical success rate of WCS implantation was close to 100%. Though WCS has relatively poor flexibility and requires rapid intraoperative catheter exchange upon release (14), experienced interventional physicians find it simple and easy to implant WCS successfully into the parent artery of BBA. Aneurysm complete occlusion and unrupture are the most important indicators to evaluate intraoperative efficacy. According to the meta-analysis, WCS treatment had a satisfying complete occlusion rate of 98.2%, and no BBA had ruptured during surgery. On the contrary, the rate of endoleak was 1.8%. Endoleak is an important issue of WCS treatment for BBA. It is extremely dangerous because blood flow from the aneurysm cavity is not smooth and will increase the internal pressure of the aneurysm, thus increasing the risk of aneurysm rupture (14). Therefore, various methods should be used during surgery to improve the complete occlusion rate and avoid endoleak. The side branch occlusion rate was 4.1%, and five cases of ophthalmic artery (OA) occlusion and two cases of anterior choroidal artery (AChA) occlusion occurred among the 102 patients. The majority of OA occlusion may not cause an important neurological dysfunction because the blood flow after OA occlusion can be compensated by the external carotid artery system due to extensive anastomosis in these two conditions (23). Nevertheless, AChA occlusion can cause catastrophic events, such as hemiparesis, hemianopsia, and hemihypesthesia (24). Although all patients with side branch occlusion were asymptomatic in all of the included studies, doctors cannot rely on luck. A pre-operative evaluation of the important side branch of the parent arteries is very important. The choice of WCS should be abandoned if the implantation leads to serious ischemic events due to the close relationship between the aneurysm and the side branch. In addition, the rate of intraoperative vasospasm in the present study was 9.2%. Though high heterogeneity (*I*^2^=77%) could make this result unreliable, vasospasm remains a significant intraoperative complication. The stimulation caused by WCS for the arterial wall is the reason for aggressive vasospasm (14). Handling includes enhancing the support by positioning the catheter, speeding up the procedure of surgery, and intravenously injecting nimodipine. If the vasospasm persists without relief, then it may increase the risk of post-operative rebleeding (15). Only one patient had a mild dissection that occurred due to the guiding catheter but disappeared at the 3-month follow-up. Dissection can be avoided through gentle manipulation during the operation (17).

In the post-operative situation, the rebleed and mortality rates were 2.2 and 1.5%, respectively. All the deceased patients experienced rebleeding. The mentioned values were comparable with the rebleed and mortality rates of stent-assisted coil embolization for saccular aneurysms (20). Meanwhile, no infarction was reported in all of the included studies. All of these findings confirmed the safety of WCS treatment for BBA. The reason for rebleeding remains uncertain but may be attributed to one of the following: (1) rupture of the WCS membrane, (2) intraoperative or postoperative endoleak (endoleak might occur after the vasospasm disappeared after surgery), and (3) angiography may reveal only a part of the lesions of BBA, and thus WCS covers only a part of the diseased area (15). To prevent rebleeding as much as possible, the development of materials, accumulation of experience, and improvement of techniques are all indispensable.

The follow-up data revealed that, after 1–36 months of follow-up, only 2 of the 95 patients developed mild recurrence, and their recurrent aneurysms remained persistent after 12 months of conservative treatment. The recurrent aneurysms were all saccular aneurysms and significantly smaller than the original BBAs (15). For the underlying reason, the WCS membrane is only adherent at several points of the alloy stent struts. After surgery, part of the WCS membrane may expand, leading to the recurrence of an aneurysm located in the central area of WCS (25). Parent artery stenosis occurred in 9.1% of the patients, possibly because of the short follow-up time. Although none of these patients had clinical symptoms, in-stent stenosis remains a problem that cannot be ignored. Mechanical injury caused by the balloon-expandable stent and resistance to antiplatelet therapy can lead to stenosis (26). In addition, chronic diseases, such as hyperlipemia, hypertension, and diabetes, are risk factors for in-stent stenosis (27, 28). Given that most of the factors mentioned are difficult to control, a regular and long-term angiography follow-up is necessary. No hemorrhage, infarction, or death was reported in all of the included studies. Ultimately, 95.7% of the patients had a good outcome, but the rest of them were in poor health before the treatment. Zhu et al. (10) conducted a meta-analysis on the efficacy of FD treatment for BBA in 2018 and found that, among the 150 patients with BBA, 83.0% had a good outcome by the same definition. Despite being limited by the nature of non-comparative studies, no statistical comparison can be performed between the two rates. Along with its high technical success rate, a high aneurysm complete the occlusion rate and a low recurrence rate, and the efficacy of WCS treatment for BBA was confirmed.

The meta-analysis of the present study achieved positive results, but there are still some limitations. As a rare disease, the characteristics of BBA vary greatly among individuals: every patient has a different condition after the BBA rupture. In addition, the application time of WCS is too short, making it difficult to conduct studies with a large sample size and control the selection bias. Randomized controlled trials or comparative studies are also difficult to perform. WCS is so expensive that some patients cannot afford it and are forced to choose other treatments, and this situation magnifies the selection bias further. Owing to the nature of non-comparative studies, this meta-analysis failed to complete the sensitivity analysis and the test for publication bias, thus affecting the authenticity of the results to a certain extent. Given that WCS has only been recently used to treat BBA, raw data available for analysis on long-term follow-up outcomes are currently lacking. Hence, the results of the present study are relatively one-sided.

## Conclusion

Although this study demonstrated that WCS could be effective and safe for BBA treatment, the available evidence is indefinite. With the continuous promotion of WCS, clinical centers should have comprehensive BBA treatment options that would allow them to choose the most appropriate treatment option in accordance with the patient's condition. The results provide a reference for clinical trials. Well-designed prospective cohort studies must be conducted for verification.

## Data availability statement

The original contributions presented in the study are included in the article/supplementary material, further inquiries can be directed to the corresponding author.

## Author contributions

The concept and design of the present study were performed by JT and ZH. The first draft of the manuscript was written by JT. Acquisition, analysis, and interpretation of data were performed by RS, SL, WF, and JS. Supervision of the study was conducted by ZH. All authors read and approved the final manuscript.
